# Heightened Prevalence of Common Hospital-Treated Infections Preceding Dementia Diagnosis with Accelerated Dementia Onset after Influenza

**DOI:** 10.14283/jpad.2024.92

**Published:** 2024-05-28

**Authors:** H. Untersteiner, R. Wurm, B. Reichardt, S. Goeschl, E. Berger-Sieczkowski, T. König, T. Parvizi, S. Silvaieh, Elisabeth Stögmann

**Affiliations:** 1 Department of Neurology, Medical University of Vienna, Waehringer Guertel 18-20, 1090 Vienna, Austria; 2 Comprehensive Center for Clinical Neurosciences and Mental Health, Medical University of Vienna, Vienna, Austria; 3 Austrian Social Health Insurance Fund, Eisenstadt, Austria

**Keywords:** Dementia, influenza, intestinal infection, sepsis, urinary tract infection

## Abstract

**Background:**

Since the beginning of Alzheimer’s disease research, the hypothesis that infections are to some extent associated with neurodegenerative processes has been tested repeatedly. Epidemiological studies on the associations between infections and dementia have reported conflicting results.

**Objectives:**

This study analyses common hospital-treated infections (herpes, influenza, intestinal infections, pneumonia, sepsis, urinary tract infections) and their association with subsequent dementia and time until dementia onset.

**Design, Setting, and Participants:**

For this nationwide population-based case-control study, the dataset of the Austrian National Health Insurance Association was used, including dementia patients (dementia cohort) and age- and gender-matched non-demented individuals (control cohort). Only subjects with data availability of at least 10 years prior to the index date (date of dementia diagnosis or date of censoring) were included.

**Measurements:**

The incidence of six common infections in older adults (herpes, influenza, intestinal infections, pneumonia, sepsis, and urinary tract infections) was analyzed over a period of 10 years before the censoring date.

**Results:**

The study population consists of 58208 subjects (29104 per study cohort), mean age: 81 years, 54% females. Patients of the dementia cohort had suffered from infections significantly more often than patients of the control cohort (6002, 20.6% vs. 4826, 16.6%; p < 0.001). Influenza, urinary tract infections, intestinal infections, and sepsis showed independent positive associations with subsequent dementia diagnosis, irrespective of other comorbidities (odds ratios: 1.26 (95% CI: 1.06–1.49), 1.23 (95% CI: 1.16–1.30), 1.16 (95% CI: 1.07–1.27), 1.17 (95% CI: 1.01–1.37), respectively). Time from infection to dementia diagnosis was shorter after influenza compared to all other infections (median: 3.4 years (95% CI: 3.1–3.7) vs. 6.6 years (95% CI: 6.4–6.8); p < 0.001).

**Conclusion:**

This is the first study to assess the association between infections and dementia over such a long minimum reporting period. These results, supported by consistent data from other epidemiological studies, emphasize the critical importance of infection prevention measures, especially for older adults. Further research is crucial to better understand the nature of the relationship between infections and dementia.

**Electronic Supplementary Material:**

Supplementary material is available in the online version of this article at 10.14283/jpad.2024.92.

## Introduction

**A**pproximately 55 million people worldwide suffer from dementia. Due to sociodemographic changes, the incidence is increasing, particularly in low- and middle-income countries (1). The most common type of dementia is Alzheimer’s disease (AD) followed by vascular dementia, Lewy body dementia, and frontotemporal dementia (2). Additionally, mixed causes are possible, most often occurring as a combination of AD and vascular pathology. Up to 40% of all dementia cases are attributed to potentially modifiable risk factors, the remaining cases are caused by unmodifiable/genetic risk factors (3). In the last decades, research has made significant progress in understanding the biomolecular pathology underlying dementia. For AD, the ATN framework has been established, which describes the consecutive occurrence of amyloid-β (Aβ) plaques (A), tau tangles (T) and neurodegeneration (N), as the cause for cognitive impairment (4). In addition to these changes, it has become increasingly apparent in recent years that neuroinflammation significantly influences disease development. This is supported by growing evidence of genetic and preclinical studies indicating an important involvement of the immune system in AD pathophysiology (5). Moreover, vascular and infectious factors are also thought to collectively influence the amyloid cascade, thus the risk of dementia and the course of the disease (6).

From the very beginning of AD research, the possibility that infections could cause AD-typical pathological changes has been explored repeatedly (7). The microbial hypothesis of AD suggests that pathogens play a causative role in disease-related neurodegenerative processes (7). There are several hypotheses to explain this relationship. For one, pathogens within the central nervous system (CNS) may trigger low-grade local neuroinflammation. While the blood-brain barrier typically shields the CNS against such detrimental factors in normal physiological conditions, age and/or inflammation processes can compromise its integrity (8). Alternatively, certain pathogens may infiltrate the CNS through other routes, such as the axonal transport utilized by the herpes simplex virus (HSV) (9). Subsequently, the low-grade local neuroinflammation contributes to increased production of Aβ, tau hyperphosphorylation, and ultimately, neurodegeneration (9). The hypothesis of direct local effects of pathogens is supported by the discovery of HSV type 1 DNA in brains and later specifically in Aβ plaques of AD patients (10, 11). More recently, a range of other pathogens, including viruses (e.g., other herpes viruses, Epstein-Barr virus, and cytomegalovirus), bacteria (e.g., chlamydia pneumoniae, Escherichia coli, and helicobacter pylori), and fungi (e.g., Saccharomyces cerevisiae, and Penicillium) have been identified in AD brains (7, 12). However, no pathogen has been universally found in all AD brains, making it unlikely that the local effects of any one specific pathogen could be the sole cause of the pathophysiological changes observed in AD (12).

Alternatively, pathogens might also enhance neuroinflammation and AD pathology without entering the CNS. By triggering the release of pro-inflammatory cytokines, such as certain interleukins (e.g. IL-6 and IL-12) and tumor necrosis factor (TNF)-α, from the periphery, chronic low-grade neuroinflammation could be induced indirectly. It has been shown that these chemokines are associated with an increased burden of Aβ, phosphorylated tau, hippocampal atrophy, and elevated risk of dementia (13, 14). This theory is in line with epidemiological study results, showing associations between dementia and non-infectious diseases that trigger systemic inflammation, like systematic rheumatoid diseases (15).

Epidemiological studies on infections and subsequent dementia risk have been conducted in the past. However, the results have been rather conflicting. Some studies were able to show associations between dementia and common infections like pneumonia and sepsis (16–23). However, other studies have presented contrasting results (24–26). Most of the published studies lack high sample sizes and long reporting periods (27). Due to the long pre-clinical phase of AD, an extended follow-up duration is needed to observe the possible effects of infections on the neurodegenerative processes.

In a large nationwide case-control study, we analyzed whether dementia patients have more frequently suffered from common hospital-treated infections (pneumonia, influenza, urinary tract infections (UTIs), intestinal infections, herpes simplex virus 1, 2 (HSV1, HSV2) and herpes zoster infection/reactivation, and sepsis) than non-dementia controls. In addition to that, the time from the different infections to dementia diagnosis was analyzed. To improve the data quality of the study, only individuals with data available for at least ten years prior to dementia diagnosis were included.

## Methods

This study retrospectively analyses a prospectively maintained dataset from the Austrian National Health Insurance Association, covering the period between 01.01.2006 and 30.06.2022. This dataset can be used for analyzing diseases treated in hospitals, as the ICD-10 codes of illnesses documented in hospital discharge letters are transmitted to the insurance association for hospital funding purposes. In total, 233660 individuals with dementia and more than twice as many nondementia controls were included in the whole dataset. This analysis is based on a sub-sample, including only subjects for whom data were collected for at least 10 years prior to the diagnosis of dementia. The period before dementia diagnosis is hereafter referred to as the reporting period. This study was approved by the Ethics Committee of the Medical University of Vienna (EK 1308/2022).

### Dementia case identification and case-control matching

Diagnosis of dementia was defined by either documentation of the dementia diagnosis in a hospital discharge letter (ICD-10 codes: F00.*, F01.*, F02.*, F03, and G30.*) or a prescription of an anti-dementia medication (rivastigmine, donepezil, galantamine, and memantine). The earliest date on which one of these conditions applied was taken as the date of the dementia diagnosis (= index date). To simplify terminology, this study cohort will hereafter be referred to as the dementia cohort.

Control subjects are randomly selected individuals, who used health care services covered by the Austrian National Health Insurance Association during the reference period, but who did not receive a diagnosis of dementia or anti-dementia treatment. Individuals in the control cohort were matched 1:1 for age at start of the reporting period and sex to individuals in the dementia cohort. For the control cohort, only data collected within the reporting period of the matched subject in the dementia cohort were considered. The censoring date corresponds to the index date in this cohort.

### Infectious diseases

For this study, the following infectious diseases were analyzed: intestinal infections, UTIs, influenza, pneumonia including aspiration pneumonia, infection/reactivation of HSV 1, 2 and herpes zoster, and sepsis. In the dataset, an infection is recorded with the corresponding ICD-10 code (see supplementary Table 1) when the disease was specified on a hospital discharge letter. Infectious diseases diagnosed within 4 weeks before the index date were not included.

### Statistical analysis

Variables are presented as absolute and relative frequencies for categorical variables, or as median with interquartile ranges for continuous variables. Differences in categorical variables between the study cohorts were analyzed using Chi^2^-tests. Differences in continuous variables were assessed using Kruskal-Wallis-test.

Odds ratios (ORs) were calculated using binary logistic regression with dementia y/n as the dependent variable. ORs are indicated with the respective 95% confidence interval (CI). Models were adjusted for all infectious diseases analyzed (= infection adjusted) or adjusted for all infectious plus common non-infectious comorbidities (= fully adjusted) including alcohol abuse, atherosclerosis, cerebrovascular disease, chronic kidney disease, chronic liver disease, chronic obstructive pulmonary disease (COPD), diabetes mellitus, hyperlipidemia, hypertension, ischemic heart disease, and obesity (ICD-10 codes for these comorbidities are listed in Supplementary Table 2). Sepsis was calculated separately in a non-adjusted model and a model adjusting only for non-infectious diseases.

The time from infection diagnosis to the onset of dementia was estimated using the Kaplan-Meier method, excluding all subjects with multiple different infections. If the same subject was diagnosed with the same infectious disease more than once, only the earliest date of diagnosis was included in this analysis. Comparisons were performed using the log-rank test.

For all tests, p < 0.05 was considered statistically significant. Calculations were performed with IBM SPSS Statistics for Windows version 29 (IBM Corp.).

### Data availability

Requests for access to the dataset should be made to Prof. Elisabeth Stögmann at elisabeth.stoegmann@meduniwien.ac.at.

## Results

### Study population

A total of 58208 subjects were included in this study, with 29104 subjects per study cohort. Out of the patients in the dementia cohort, 24834/29104 (85.3%) received antidementia medications, 1585/29104 (5.4%) were diagnosed with dementia on hospital discharge letter, and 2685/29104 (9.2%) met both conditions. At the index date, the median age was 81 years (76–86), with 15772 (54.2%) individuals in the dementia cohort and 15758 (54.1%) in the control cohort being older than 80 years. In both cohorts, 17792/29104 (61.1%) subjects are female. The median reporting period for both study cohorts amounts to 11.0 years (10.5–11.6). All analyzed non-infectious comorbidities were significantly more frequent in the dementia than in the control cohort, (p<0.001). The most pronounced group differences were observed in hypertension (14363/29104, 49.4% vs. 12586/29104, 43.2%) and cerebrovascular disease (4009/29104, 13.8% vs. 2918/29104, 10.0%). Details of both study cohorts are shown in Table 1.

**Table 1:** Description of the study cohort. Data is displayed as absolute and relative numbers, median and interquartile range. For better gradation, also p-values <0.001 are indicated in full. COPD, chronic obstructive pulmonary disease

	**Dementia cohort (n=29104)**	**Control cohort (n=29104)**	**p-value**
Age at start of reporting period	70 (65–75)	70 (65–75)	1.000
Age at index date	81 (76–86)	81 (76–86)	0.834
Age at index date: sub-groups			0.907
≤80	13332 (45.8%)	13346 (45.9%)	
≤80	15772 (54.2%)	15758 (54.1%)	
Females	17792, 61.1%	17792, 61.1%	1.000
Reporting period [years]	11.0 (10.5–11.6)	11.0 (10.5–11.6)	0.984
Non-infectious comorbidities			
Alcohol abuse	560 (1.9%)	290 (1.0%)	1.07E-20
Atherosclerosis	1637 (5.6%)	1458 (5.0%)	9.44E-4
Cerebrovascular disease	4009 (13.8%)	2918 (10.0%)	2.52E-44
Chronic kidney disease	2813 (9.7%)	2538 (8.7%)	7.98E-5
Chronic liver disease	1782 (6.1%)	1574 (5.4%)	2.17E-4
COPD	1789 (6.1%)	1591 (5.5%)	4.50E-4
Diabetes	4367 (15.0%)	3494 (12.0%)	3.42E-26
Hyperlipidaemia	6494 (22.3%)	5365 (18.4%)	3.33E-31
Hypertension	14363 (49.4%)	12586 (43.2%)	2.24E-49
Ischemic heart disease	4831 (16.6%)	4351 (14.9%)	4.81E-8
Obesity	1776 (6.1%)	1622 (5.6%)	0.006

### Infections in both study cohorts

In total, 10828/58208 (18.6%) individuals were diagnosed with one of the following infections during the reporting period: influenza (568/58208, 1.0%), UTIs (5637/58208, 9.7%), intestinal infections (2358/58208, 4.1%), herpes including HSV1, HSV2 and herpes zoster (833/58208, 1.4%), and pneumonia (4094/58208, 7.0%). Overall, 18914 infections were diagnosed, 10702 (56.6%) in the dementia cohort and 8212 (43.4%) control cohort. In total, 2297/58208 (3.9%) patients had more than one type of infection.

Of the dementia cohort, 6002/29104 (20.6%) subjects were diagnosed with at least one infection. This percentage is significantly higher than that of the control cohort, where 4826/29104 (16.6%) individuals had an infection (p < 0.001), see Table 2. When comparing subjects with infections, individuals in the dementia cohort significantly more often had multiple infections (including different types of infections or the recurrence of the same type of infection) compared to single infections than in the control cohort (2160/6002, 36.0% vs. 1631/4826, 33.8%, p = 0.017), see Table 2.

**Table 2:** Absolute and relative numbers of infections overall and specific infections in both study cohorts. UTI, urinary tract infection

	**Dementia cohort (n=29104)**	**Control cohort (n=29104)**	**p-value**
Infections overall	6002 (20.6%)	4826 (16.6%)	<0.001*
Multiple infections	2160/6002 (36.0%)	1631/4826 (33.8%)	0.017 t
Influenza	334 (1.2%)	234 (0.8%)	<0.001
UTI	3221 (11.1%)	2416 (8.3%)	<0.001
Intestinal infection	1337 (4.5%)	1021 (3.5%)	<0.001
Herpes	461 (1.6%)	372 (1.3%)	0.002
Pneumonia	2202 (7.6%)	1892 (6.5%)	<0.001
Sepsis	388 (1.3%)	296 (1.0%)	<0.001

* This p-value of <0.001 corresponds to 5.35E-36; † Significance indicates the difference in the frequency of multiple vs. single infections between both cohorts.

**Figure 1:**
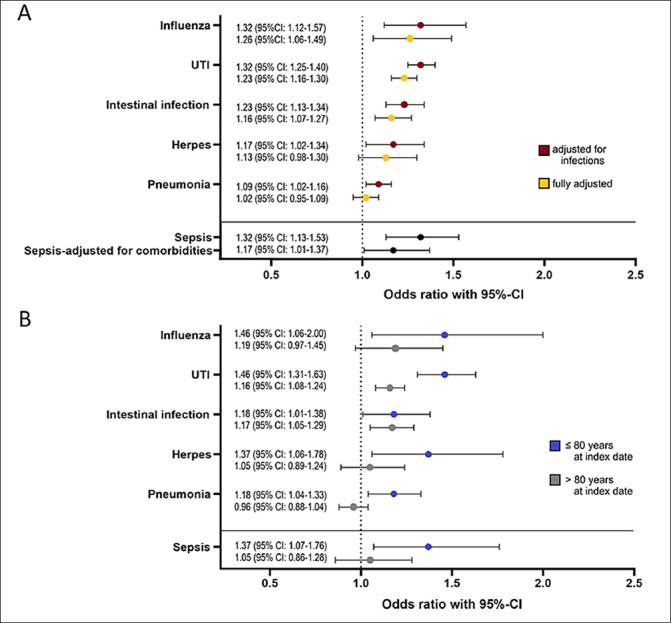
A: Logarithmic regression analysis was applied to assess the relationship between dementia and prior infections (intestinal infection, urinary tract infection (UTI), pneumonia, influenza, herpes, and sepsis). The figure displays the odds ratios (ORs) for these six infections. The infection-adjusted model includes all infections except sepsis and the fully adjusted model additionally includes non-infectious comorbidities (alcohol abuse, atherosclerosis, cerebrovascular disease, chronic kidney disease, chronic liver disease, chronic obstructive pulmonary disease, diabetes mellitus, hyperlipidaemia, hypertension, ischemic heart disease, obesity). ORs for sepsis are calculated without adjustment and with a model adjusting only for non-infectious comorbidities. B: ORs were calculated separately for two different age groups (≤80 years and >80 years at the index date) with the fully adjusted model (influenza, UTIs, intestinal infection, herpes, pneumonia) and the model correcting only for non-infectious comorbidities (sepsis)

ORs calculated with the infection-adjusted model indicate a significant association between all infections and subsequent dementia: influenza 1.32 (95% CI: 1.12–1.57), UTI 1.32 (95% CI: 1.25–1.40), intestinal infection 1.23 (95% CI: 1.13–1.34), herpes 1.17 (95% CI: 1.02–1.34), pneumonia 1.09 (95% CI: 1.02–1.16). In the fully adjusted model, patients in the dementia cohort were significantly more likely to have had influenza, UTIs, and intestinal infections than patients in the control cohort. The ORs are 1.26 (95% CI: 1.06–1.49) for influenza, 1.23 (95% CI: 1.16–1.30) for UTIs, 1.16 (95% CI: 1.07–1.27) for intestinal infections, see Figure 1A for details.

Age-stratified analyses, categorizing patients into two groups (≤80 years and >80 years at the index date) reveal that individuals diagnosed with dementia at a younger age faced a higher risk of having had an infection within the reporting period. In the younger age group, even in the fully adjusted model, all infections were significantly associated with dementia, with ORs of 1.46 (95% CI: 1.31–1.63) for UTIs, 1.46 (95% CI: 1.06–2.00) for influenza, 1.37 (95% CI: 1.06–1.78) for herpes, 1.18 (95% CI: 1.01–1.38) for intestinal infections, and 1.18 (95% CI: 1.04–1.33) for pneumonia. In the older age group, intestinal infections (OR: 1.17, 95% CI: 1.05–1.29) and UTIs (OR: 1.16, 95% CI: 1.08–1.24) were significantly more common in the dementia cohort (see Figure 1B).

Sub-analyses were performed to assess differences between different types of pathogens causing intestinal infections. Bacterial intestinal infections were diagnosed in 451 patients, viral infections in 404 patients, and protozoa in 8 patients. The majority of patients (n = 1642) had an intestinal infection of unspecified origin. Patients in the dementia cohort were significantly more likely to have an intestinal infection of unspecified origin (OR: 1.26, 95% CI: 1.14–1.39) than patients in the control cohort. However, there was no significant difference in the incidence of bacterial, viral or protozoal infections between the two cohorts.

Recurrent UTIs were more strongly associated with dementia than singular UTIs (singular vs. none: OR: 1.20, 95% CI: 1.12–1.28; recurrent vs. none: OR: 1.27, 95% CI: 1.14–1.42).

For other infections, there was no increase in dementia risk due to recurrence. There was no significant difference in hospital stay duration due to any infection between both cohorts (dementia cohort: 9 days (6–14), control cohort: 9 days (5–14)). When calculated separately for each infection, only the number of days spent in the hospital for intestinal infections was significantly higher in patients in the dementia cohort (7 (4–11) vs. 6 (3–10) days, p = 0.002).

### Sepsis

In our cohort, 388/29104 (1.3%) of the dementia cohort patients and 296/29104 (1.0%) of the controls suffered from sepsis in the reporting period, resulting in an unadjusted OR of 1.32 (95% CI: 1.13–1.53). After adjustment for common non-infectious comorbidities, the OR remained significant (1.17, 95% CI: 1.01–1.37), see Figure 1A. Again, the OR was higher (1.37, 95% CI: 1.07–1.76) when only patients with dementia onset ≤80 years were included in the comorbidity-adjusted model, see Figure 1B. Whether patients had suffered recurrently from sepsis or not had no significant effect on the risk of dementia and neither did the number of days of hospitalization.

### Time from infection to dementia

**Figure 2:**
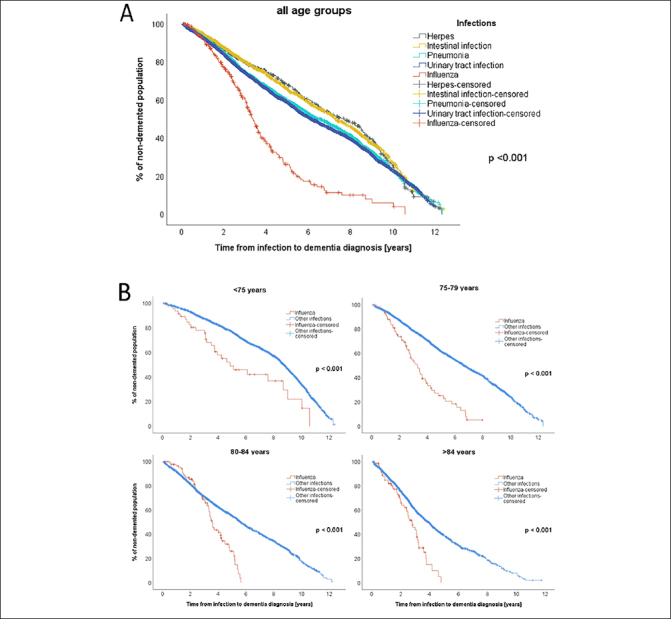
A: Kaplan-Meier survival analysis used for calculating times between infections (pneumonia (n=2541), influenza (n=295), intestinal infection (n=1338), urinary tract infection (n=3852), herpes (n=505)) and subsequent dementia diagnosis. Patients with multiple different infections (n=2297) were excluded from this analysis. In cases where one infection recurred, the age at the first episode was considered. B: Kaplan Meier survival analysis stratified by the age groups at time of infection: <75 years (influenza: n=49, other infections: n= 2305), 75–79 years (influenza: n=77, other infections: n=2122), 80–84 years (influenza: n= 91, other infections: n=1979), >84 years (influenza: n= 78, other infections: n= 1830)

To analyze whether time between infection and the dementia onset differed between infection types, Kaplan-Meier survival analyses were performed (Figure 2A). The shortest time from infection to dementia diagnosis was found for influenza (3.4 years (95% CI: 3.1–3.7)), followed by UTIs (6.2 years (5.9–6.5)), pneumonia (6.6 years (95% CI: 6.2–7.0)), intestinal infections (7.3 years (95% CI: 6.9–7.8)), and herpes (7.7 years (95% CI: 6.8–8.7)). The difference in time from infection to dementia diagnosis was significant between all infections (p < 0.001). This calculation was repeated comparing only influenza to all other infections (influenza to dementia: 3.4 years (95% CI: 3.1–3.7) vs. other infections to dementia: 6.6 years (95% CI: 6.4–6.8); p < 0.001).

To minimize the influence of age, the same calculation was performed again, stratified by age at infection (age groups: <75 years, 75–79 years, 80–84 years, ≥85 years). The difference between the infections remained significant (p < 0.001) in all sub-analyses (see Figure 2B). Among the individuals below the age of 75, the median duration between influenza infection and dementia diagnosis was 4.9 years (95% CI: 2.6–7.1), while for other infections, it was 8.8 years (95% CI: 8.6–8.9). For those aged 75–79 years at the time of infection, dementia was diagnosed at a median of 3.3 years (95% CI: 2.6–4.0) after influenza infection and 6.7 years (95% CI: 6.3–7.0) after other infections. In the age group 80–84 years, the median time from influenza to dementia diagnosis was 3.6 years (95% CI: 3.3–3.9) compared to 5.7 years (95% CI: 5.3–6.0) for other infections. Among the individuals aged ≥85, dementia was diagnosed after a median time of 2.8 years (95% CI: 2.2–3.3) after influenza and 4.0 years (95% CI: 3.7–4.2) after other infections.

## Discussion

The hypothesis that infections may influence the development of dementia has been studied for decades (7). However, the exact mechanism behind this has not yet been understood. Epidemiological studies are still sparse and results inconsistent, which emphasizes the need for increased research efforts. In this study, we analyzed the association between common hospital-treated infections and subsequent dementia, using data from the period of at least ten years before dementia diagnosis.

Overall, patients in the dementia cohort were significantly more frequently hospitalized for infections than the control cohort. When correcting for non-infectious comorbidities, there is still an independent association between UTIs, influenza, and intestinal infections and subsequent all-cause dementia. Additionally, instances of multiple infections were more prevalent within the dementia cohort compared to singular infections. These findings suggest that infections influence the development of dementia and hint at the presence of a dose-response relationship.

In other epidemiological studies, UTIs, influenza, and intestinal infections were rarely analyzed. However, most existing data are in line with our results, showing significant associations of dementia with prior UTIs and influenza (18, 21, 22, 28). Two studies report no independent relationship between intestinal infections or influenza and the risk of dementia (26, 28). This discrepancy could be attributed to the study’s smaller sample size or the fact that only infections treated at primary care facilities were considered, which are presumably less severe and therefore cause less pronounced systemic inflammation, possibly not as strongly correlated with neurodegeneration (29).

Pneumonia and herpes were associated with subsequent dementia in the model correcting only for other infections, but not in the fully adjusted model. These two infections and their association with dementia have been studied more extensively in the past than the other infections analyzed here. While some epidemiological studies have identified a correlation between these infections and dementia (20, 22, 23, 30–34), conflicting findings, including those from a Mendelian randomization study, cast doubt on the association (35–38). The reasons behind these conflicting results remain unclear. Potential factors may include dataset disparities (such as differences in the origin of the dataset, follow-up duration, or study samples), or differences in the methodologies applied (e.g., the extent of adjustments of regression models).

In this study, we made diligent corrections with a focus on comorbidities. These were carefully chosen to minimize confounding bias, as they are suspected risk factors for both infections and dementia. Of note, the difference between the two study cohorts in the occurrence of non-infectious diseases was less pronounced than the difference in infections, except for hypertension and cerebrovascular disease. In our opinion, this further highlights the strong association between infections and dementia.

The association of sepsis was calculated in a separate model since the condition is often caused by one of the other analyzed infections. The study results show an independent association with subsequent dementia. This finding is consistent with most other studies showing an increased risk of dementia following sepsis (16, 17, 19, 21).

The homogeneous extended reporting period of patients allowed an analysis of the temporal relationship between infections and the onset of dementia. KaplanMeier survival analyses showed that there is a significant difference in the time from infection to diagnosis of dementia, depending on infection type. It is particularly striking that the time from influenza to dementia diagnosis was significantly shorter compared with the other infections. This indicates that influenza - more than other infections - is associated with a higher short-term risk of dementia. To reduce a potential confounding effect of age at infection, age-stratified analyses were performed. The difference remained significant in all sub-analyses with different age ranges. To our knowledge, this result has not been reported before, making it hard to interpret, however, thus even more interesting. The mechanism by which the influenza virus influences the pathophysiology of dementia might differ from other pathogens. Because of the brief time interval between influenza and the onset of dementia, it cannot be excluded that the association is caused by an inverse relationship, i.e. frailty in the preclinical phase of AD could render patients more susceptible to certain diseases. However, it seems unlikely that frailty before dementia onset would make patients specifically more vulnerable to influenza (as opposed to the other infections we analyzed).

Interestingly, in our study, the association between infections and dementia was more pronounced in patients with a younger age at the onset of dementia. This finding contrasts with the results of another study that reported higher associations between infections and dementia at higher age (+90 years) (21). Further investigation would be needed to assess the influence of age in this context.

Intestinal infections of unspecified origin occurred significantly more often in the dementia cohort than in the control cohort, before the index date. No differences were observed for intestinal infections due to bacteria, viruses, or protozoa between both cohorts. This is presumably due to the lower sample size.

For UTIs, recurrent infections were more strongly associated with dementia than a singular UTI. This is in line with other epidemiological data (18). Whether there is a causal reason why recurrent episodes of other infections do not have this effect, or whether this is only due to the small sample size, will require further research.

A longer hospital stay following intestinal infections is associated more strongly with subsequent dementia risk, possibly indicating disease severity. However, other contributing factors cannot be definitively excluded.

We believe that this work makes a valuable contribution to further exploring the complex connections between infections and dementia at a clinical epidemiological level. To our knowledge, there is no existing study on this topic with such a large dataset and over such an extended minimum observational period. For many years, discussions have revolved around the associations between different infections, like herpes simplex virus, and dementia, yet, without reaching a conclusive outcome. In our study, we were unable to identify significant independent correlations in this regard. However, we did observe a markedly increased prevalence of other infections, such as influenza, intestinal infections, UTIs, and sepsis, even after rigorous adjustment for additional factors.

Interestingly, studies show that vaccinations have a protective role regarding dementia risk (39–41). Notably, this phenomenon is not specific to a particular vaccination but has been observed with various types. Similarly, in this study, the correlation between infections and dementia was not limited to one pathogen. This could suggest that the systemic inflammation process, triggered by diverse pathogens may have implications on neurodegeneration rather than the effects of specific pathogens. This hypothesis aligns with the dose-response relationship between multiple different infections and subsequent dementia that we observed, as well as the stronger correlation in individuals with an earlier onset of dementia. The role of infections/systemic inflammation in the context of the ATN framework remains unclear. In murine models, systemic inflammation induced by lipopolysaccharide, polyinosinic-polycytidylic acid, and TNFα has been shown to increase Aβ burden (42). However, the association between infections and dementia seems to be stronger when the period between both events is shorter (21), which might suggest that inflammation may have a stronger impact on a later stage of the ATN framework.

The gut-brain axis, and thus the gut-microbiome-brain axis, may also play an important role in this relationship, particularly in the case of intestinal infections. On one hand, there is a bidirectional relationship between infections and the gut microbiome, because infections or anti-infectious treatments like antibiotics can cause gut dysbiosis, and an unbalanced microbiome increases the risk of infection (43). On the other hand, literature provides evidence linking the development of AD to changes in the gut microbiome (44).

In our study, we observed a notable temporal proximity between influenza and the onset of dementia - a relationship that, to our knowledge, has not been previously reported. Studies have described a post-acute infection syndrome following influenza (45). This condition, also referred to as “long-flu”, has been linked to neurocognitive decline including memory loss (46). Notably, the more common long-COVID condition is associated with increased levels of neurofilament light chain (NfL) and glial fibrillary acidic protein (GFAP) – both recognized biomarkers for AD (47). While there is no epidemiological data linking long-COVID to a higher risk of dementia available yet, the post-acute infection syndrome could offer a plausible explanation for the short time interval between influenza and the onset of dementia.

### Limitations

This study is limited by the absence of diagnostic data to validate the different diagnoses due to the use of an insurance-based dataset. Nevertheless, it is important to note that the ICD-10 codes that were used for the analyses are exclusively assigned by physicians in Austria. For the dementia diagnosis, we used anti-dementia treatment in addition to ICD-10 codes as a proxy for dementia. This is due to the reason that dementia diagnoses are not always recorded in the hospital discharge letter. The prescription of these medications is limited to patients diagnosed with AD by a specialist, i.e. neurologist or psychiatrist. Documentation of disease severity is required for prescription and follow-ups are needed at least biannually, making it a good proxy for dementia diagnosis, which was successfully used to identify cases in other studies (48). The role of vaccinations could not be analyzed with this dataset. It cannot be excluded, that vaccinations have a confounding effect on the association between infections and dementia. Another limitation is that reverse causality cannot be ruled out, although the median time between infection and dementia onset was relatively long (up to 7 years depending on the infection).

## Conclusion

In conclusion, our study reveals a higher prevalence of common infections including influenza, UTIs, intestinal infections, herpes, pneumonia, and sepsis in individuals with dementia compared to age- and sex-matched controls within 10 years preceding the dementia diagnosis. Notably, influenza, UTIs, and intestinal infections show an independent association with subsequent dementia, even after adjusting for other non-infectious comorbidities. This implies an elevated risk of dementia development in the years following these infections. The association was more pronounced in subjects who were diagnosed with dementia at a younger age. Remarkably, the time interval between infection and dementia diagnosis was significantly shorter after influenza compared to other infections. This difference remained significant even in age-stratified sub-analyses. To our knowledge, this is the first study to assess infections and risk of dementia with such a long minimum reporting period.

## Electronic supplementary material

Supplementary material, approximately 18.3 KB.

## Abbreviations

AD: Alzheimer’s disease
Aβ: amyloid-β
CNS: central nervous system
COPD: chronic obstructive pulmonary disease
CI: confidence interval
GFAP: glial fibrillary acidic protein
HSV: herpes simplex virus
IL: interleukins
NfL: neurofilament light chain
OR: odds ratio
TNF: tumor necrosis factor
UTIs: urinary tract infections
