# Supplementary material for: Heightened Prevalence of Common Hospital-Treated Infections Preceding Dementia Diagnosis with Accelerated Dementia Onset after Influenza
Source: J Prev Alzheimers Dis. 2024 May 28;11(5):1445–54. doi: 10.14283/jpad.2024.92 (PMC11436395; doi:10.14283/jpad.2024.92)
Supplement: Supplementary file 1 — Supplementary material, approximately 18.3 KB. [file 42414_2024_92_MOESM1_ESM.docx]

**Supplementary tables:**

| Infections | ICD-10 code |
| --- | --- |
| Intestinal infections | A00.*, A01.*, A02.*, A03.*, A04.*, A05.*, A06.*, A07.*, A08.*, A09.* |
| Urinary tract infections | N39.0 |
| Influenza | J10.*, J11.* |
| Pneumonia | J12.*, J13, J14, J15.*, J16.*, J17.*, J18.*, J69.* |
| Herpes simplex virus type 1 and 2/herpes zoster | A60.*, B00.*, B02.* |
| Sepsis | A26.7, A28.2, A32.7, A39.2, A39.4, A40.*, A41.*, A42.7, A54.8, B00.7, B34.8, B37.7, B44.7, B48.8, B60.8 |

**Supplementary Table 1:** ICD-10 codes for all analysed infections

| Non-infectious comorbidity | ICD-10 code |
| --- | --- |
| Diabetes mellitus | E10.*, E11.* |
| Hyperlipidaemia | E78.* |
| Alcohol abuse: | F10.* |
| Hypertension | I10.* |
| Ischemic heart disease | I25.* |
| Cerebrovascular disease | I60.*, I61.*, I62.*, I63.*, I64, I65.*, I66.*, I67.* |
| Atherosclerosis | I70.* |
| COPD | J44.* |
| Chronic liver disease | K70.*, K71.*, K72.0, K72.1, K72.9, K73.*, K74.0, K74.1, K74.2, K74.3, K74.4, K74.5, K74.6, K75.*, K76.* |
| Chronic kidney disease | N18.* |
| Obesity | E66.* |

**Supplementary Table 2:** ICD-10 codes for non-infectious comorbidities. COPD, Chronic Obstructive Pulmonary Disease.
